# Early Initiation of Sacubitril/Valsartan in Intracerebral Hemorrhage With Refractory Hypertension and Chronic Heart Failure: A Case Report

**DOI:** 10.7759/cureus.98919

**Published:** 2025-12-10

**Authors:** Takeru Nishino, Ayumu Yamaoka, Katsuya Komatsu, Yukinori Akiyama, Nobuhiro Mikuni

**Affiliations:** 1 Department of Neurosurgery, Sapporo Medical University, School of Medicine, Sapporo, JPN

**Keywords:** cerebral hemorrhage, chronic heart failure, perioperative blood pressure management, refractory hypertension, sacubitril/valsartan

## Abstract

Intracerebral hemorrhage (ICH) is a devastating type of stroke associated with high mortality and morbidity, and its management becomes more challenging when complicated by severe comorbidities such as chronic heart failure. Sacubitril/valsartan, an angiotensin receptor-neprilysin inhibitor, was originally developed for chronic heart failure but has also demonstrated potent blood pressure-lowering effects. In Japan, sacubitril/valsartan has had an approved indication for hypertension, supported by multiple clinical trials, including the phase III study. However, its application in the acute management of ICH remains extremely limited. We report the case of a man in his 50s with right putaminal hemorrhage complicated by refractory hypertension and chronic heart failure. On admission, his blood pressure remained elevated despite continuous intravenous nicardipine infusion at 11 mg/h and additional nitroglycerin. He subsequently required a combination of five antihypertensive agents. Following surgical evacuation of the hematoma, we transitioned his therapy to oral agents on day two. By initiating and rapidly titrating sacubitril/valsartan, we achieved effective blood pressure control without causing hypotension. In addition, the patient’s significantly elevated N-terminal pro-B-type natriuretic peptide level decreased from 1823 to 272 pg/mL, accompanied by dramatic improvements in cardiac size and pulmonary congestion. The clinical course suggests that sacubitril/valsartan offers a unique dual benefit in this high-risk patient population: it not only provides effective blood pressure control but also delivers crucial cardioprotective effects. This report highlights its potential as a game-changing therapeutic option, particularly during the critical transition from intravenous to oral therapy in acute ICH.

## Introduction

Intracerebral hemorrhage (ICH) remains a devastating form of stroke, with reported mortality rates of 30% to 40% and substantial risk of enduring neurological deficits [1]. The presence of severe comorbidities such as heart failure further complicates acute-phase management and worsens the overall prognosis [2]. Sacubitril/valsartan, an angiotensin receptor-neprilysin inhibitor, was originally developed as a treatment for chronic heart failure but has recently gained attention for its blood pressure-lowering effects [3]. In Japan, sacubitril/valsartan has had an approved indication for hypertension since September 2021, supported by multiple clinical trials, including the phase III study [4,5]. The 2025 Japanese Society of Hypertension Guidelines also mention sacubitril/valsartan as a therapeutic option for selected hypertensive patients, particularly those with concomitant cardiac disease [6]. However, its use in the acute antihypertensive management of stroke, particularly ICH, remains very limited. Here, we present a case of ICH complicated by refractory hypertension and chronic heart failure, in which early initiation of sacubitril/valsartan contributed to favorable cardiac recovery.

## Case presentation

**Figure 1:**
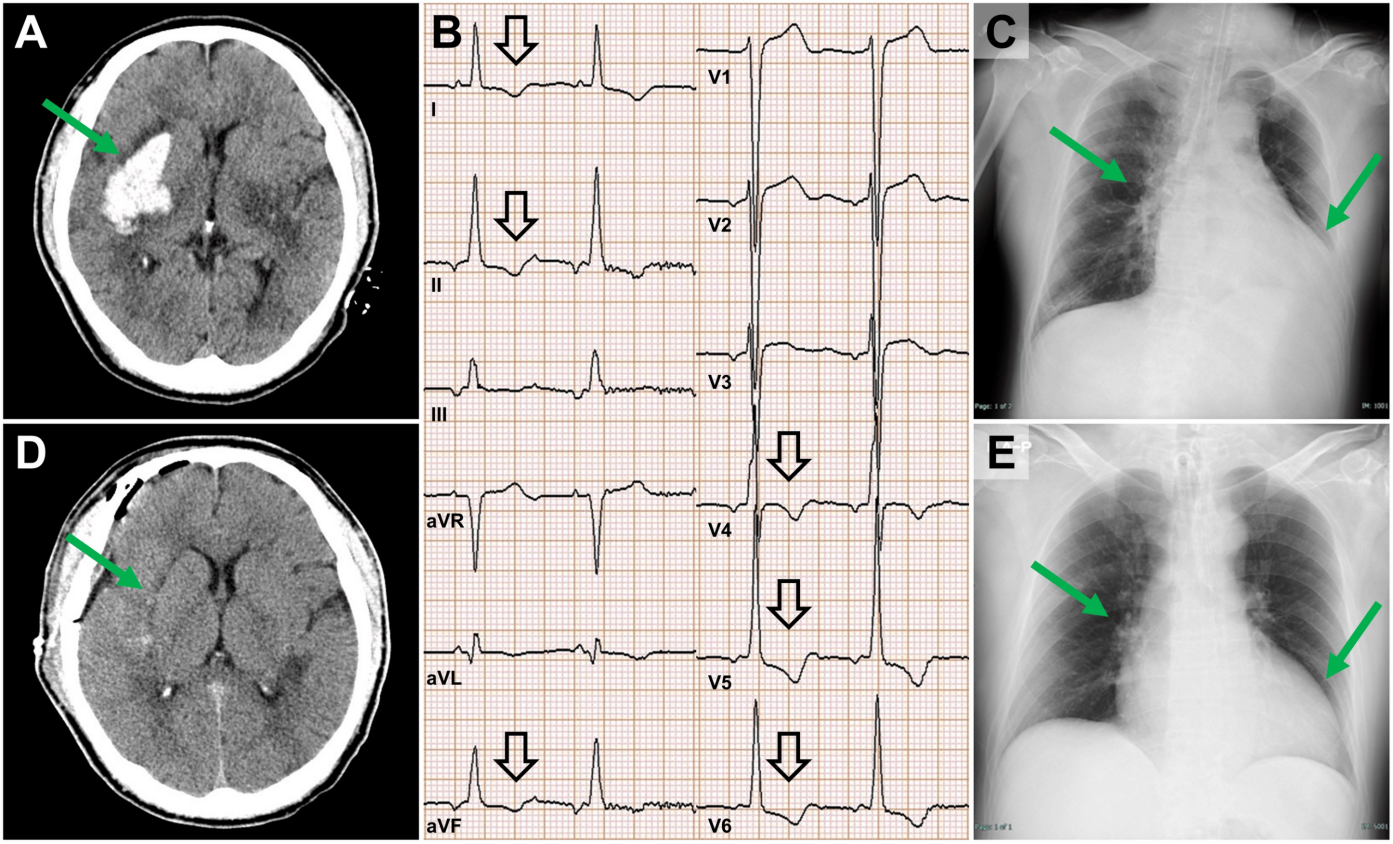
Neuroimaging and chest radiographic findings (A) Initial non-contrast CT showing a right putaminal hemorrhage. (B) Electrocardiogram on admission demonstrating ST-segment depression in leads I, II, aVF, and V4–V6. (C) Chest X-ray on admission showing cardiomegaly (cardiothoracic ratio 65%) and pulmonary vascular congestion. (D) Postoperative CT confirming near-complete hematoma evacuation (approximately 98% removed). (E) Chest X-ray at discharge showing improvement of cardiomegaly (cardiothoracic ratio 55%) and resolution of pulmonary congestion.

A man in his 50s was transferred to our hospital for surgical intervention of a right putaminal hemorrhage. He had no past medical history and had not been receiving regular medical care. Upon arrival, his level of consciousness was a Glasgow Coma Scale score of 10 (E2V2M6). His vital signs were as follows: respiratory rate, 15 breaths/min; blood pressure, 177/79 mmHg (with continuous intravenous infusion of nicardipine at 11 mg/h); heart rate, 79 bpm; and body temperature, 36.8°C. Oxygen saturation on pulse oximetry was 99% with 5 L/min oxygen. Neurological examination revealed no anisocoria or conjugate eye deviation. Manual muscle testing showed a grade of 1/5 in the left upper limb and 2/5 in the left lower limb. Head CT revealed a right putaminal hemorrhage measuring approximately 33 mL, a volume that met the surgical indication criteria [7]. (Figure 1A). Contrast-enhanced head CT showed no abnormal vascular findings, consistent with a diagnosis of hypertensive ICH. The degree of hemiparesis was more severe than expected for the hematoma size, raising concern for compression of the pyramidal tract or deep perforating arteries. Therefore, surgical evacuation was considered appropriate. On the other hand, electrocardiography revealed ST-segment depression in leads I, II, aVF, and V4-V6 (Figure 1B). A chest X-ray showed an increased cardiothoracic ratio of 65% and enhanced pulmonary vascular markings (Figure 1C). Blood tests revealed a troponin T level of 0.042 ng/mL, creatine kinase of 104 IU/L, creatine kinase-MB isoenzyme of 3.1 IU/L, and N-terminal pro-B-type natriuretic peptide (NT-proBNP) levels of 1823 pg/mL (Table 1). Transthoracic echocardiography demonstrated an interventricular septal thickness of 13 mm and posterior wall thickness of 15 mm at end-diastole, a left ventricular end-diastolic volume of 185 mL, a left ventricular end-systolic volume of 93 mL, and a left ventricular ejection fraction of 50%. Differential diagnoses included Takotsubo cardiomyopathy and coronary artery disease; however, there were no signs of heart failure or any abnormalities in hemodynamics. An emergency craniotomy and hematoma evacuation were performed via the trans-sylvian approach. Postoperative head CT confirmed that approximately 98% of the hematoma had been removed (Figure 1D).

**Table 1:** Laboratory findings on admission

Laboratory test	Result	Reference range
Hematology		
White blood cell count (×10^3^/µL)	10.6	3.3-8.6
Hemoglobin (g/dL)	13.7	13.7-16.8
Hematocrit (%)	42.1	40.7-50.1
Red blood cell count (×10^6^/µL)	4.70	4.35-5.55
Platelet count (×10^3^/µL)	245	158-348
Electrolytes / Metabolic Panel		
Sodium (mmol/L)	141	138-145
Potassium (mmol/L)	3.2	3.6-4.8
Chloride (mmol/L)	104	101-108
Calcium (mg/dL)	8.8	8.8-10.1
Magnesium (mg/dL)	2.0	1.8-2.3
Glucose (mg/dL)	115	73-109
Renal Function		
Blood urea nitrogen (mg/dL)	14.0	8–20
Creatinine (mg/dL)	0.88	0.65-1.07
Liver Function		
AST (U/L)	18	30-13
ALT (U/L)	19	10-42
ALP (U/L)	108	38-113
Total bilirubin (mg/dL)	1.0	0.4-1.5
Inflammatory Markers		
C-reactive protein (mg/dL)	1.64	<0.14
Coagulation		
PT-INR	1.0	0.85-1.15
APTT (sec)	27.2	26.9-38.1
Fibrinogen (mg/dL)	447	200–400
D-dimer (µg/dL)	0.6	<1.0
Cardiac Markers		
Troponin T (ng/mL)	0.042	<0.014
CK (U/L)	104.0	59-248
CK-MB (ng/mL)	3.1	<5
NT-proBNP (pg/mL)	1823.0	<125

The patient was admitted to the ICU postoperatively. Blood pressure was controlled with a target systolic level below 140 mmHg under continuous arterial pressure monitoring. On day two, antihypertensive therapy was transitioned from intravenous to oral medications. Sacubitril/valsartan was selected and initiated at 100 mg once daily. The dose of sacubitril/valsartan was titrated upward each day and reached 400 mg on day fpur. Despite these adjustments, the patient’s hypertension remained refractory, necessitating the addition of a calcium channel blocker, doxazosin, bisoprolol, and esaxerenone. The patient remained in the ICU until day seven under continuous arterial pressure monitoring, and his mean arterial pressure was maintained above 70 mmHg. Antihypertensive therapy via intravenous infusion was gradually tapered starting on day eight and discontinued on day 10. Subsequent blood pressure control was successfully achieved.

**Figure 2:**
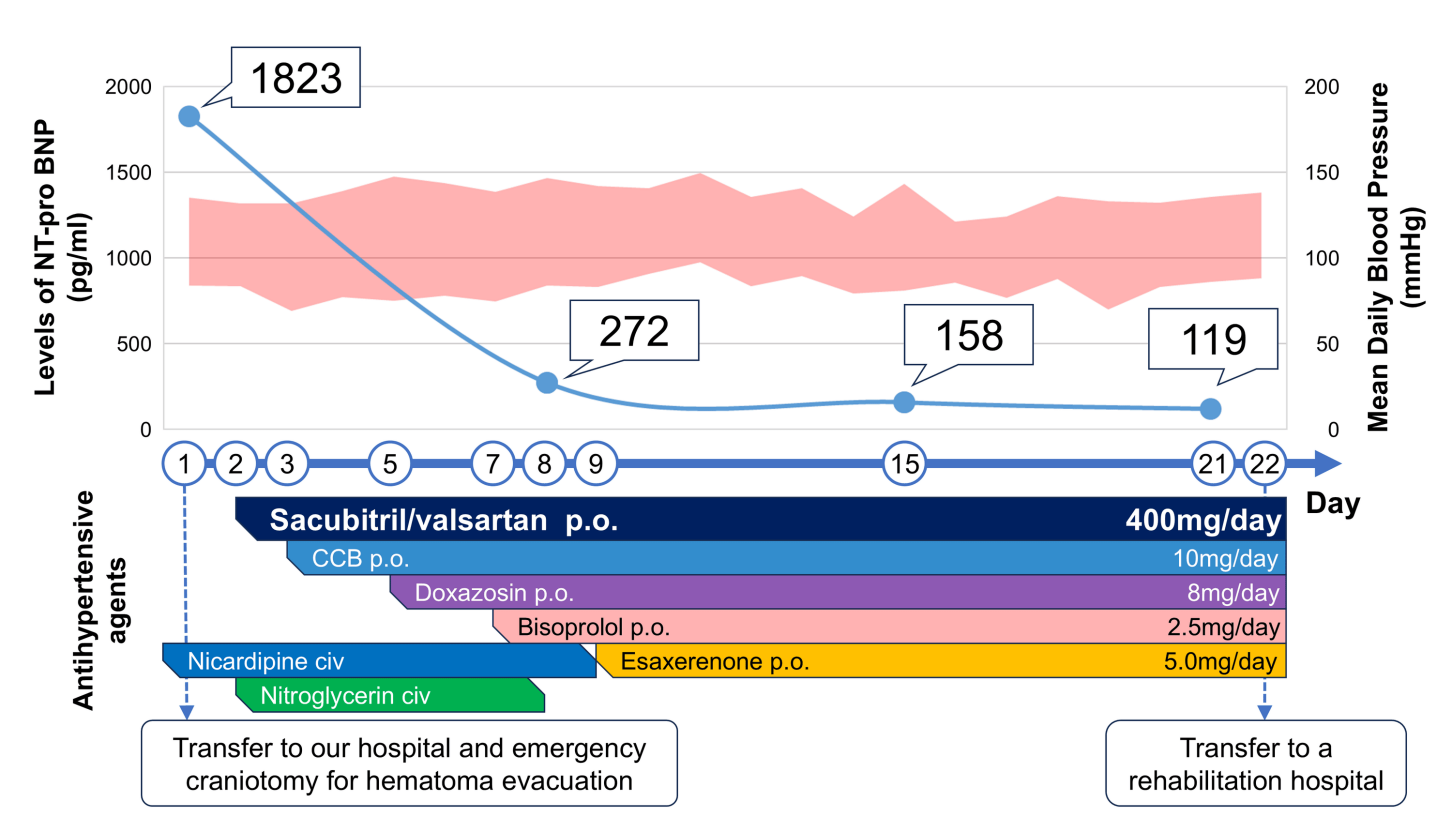
Clinical course of NT-proBNP, blood pressure, and antihypertensive therapy Serial NT-proBNP levels, mean daily blood pressure, and timing of intravenous and oral antihypertensive therapy during hospitalization. NT-proBNP was markedly elevated at admission (1823 pg/mL), declined to 272 pg/mL by day 8, and fell further to 119 pg/mL by day 21 after sacubitril/valsartan initiation, indicating sustained improvement in cardiac function. Blood pressure control was achieved in parallel with the stepwise introduction of multiple antihypertensive agents. The dosages shown for antihypertensive agents represent their final doses during the hospitalization. NT-proBNP: N-terminal pro–B-type natriuretic peptide

Regarding the management of cardiac dysfunction, strict fluid control was maintained to achieve euvolemia, which was monitored through oxygen requirements, NT-proBNP levels, body weight, and chest radiography. There was no marked increase in oxygen requirements. The patient was extubated on day three but required reintubation due to dysphagia caused by pseudobulbar palsy, and a percutaneous tracheostomy was performed on day four. He was successfully weaned from mechanical ventilation on day five. Oxygen requirements then gradually decreased, and supplemental oxygen was discontinued on day 14. NT-proBNP levels were measured weekly and showed a marked decline, from 1,823 pg/mL at admission to 272 pg/mL on day eight, 158 pg/mL on day 15, and 119 pg/mL on day 21 (Figure 2). His body weight was 95.8 kg on ICU admission and did not increase during the course of management, measuring 94.8 kg at ICU discharge on day seven. Chest radiographs were obtained daily while the patient was in the ICU and then weekly thereafter. They demonstrated progressive improvement beginning on postoperative day 8, with the cardiothoracic ratio improving to 55% by discharge and pulmonary vascular congestion resolving (Figure 1E). No acute decompensated heart failure was observed during the perioperative period. Postoperative evaluation suggested hypertensive heart disease as the underlying cause of cardiac dysfunction and left ventricular hypertrophy.

In the ICU, the patient received only bedside rehabilitation. He was discharged from the ICU on day seven, and comprehensive rehabilitation was initiated on day eight. The patient’s activities of daily living gradually improved, and he was able to mobilize with assistance using a wheelchair. On day 21, the patient was transferred to a rehabilitation facility for continued recovery.

## Discussion

This case emphasizes two important clinical considerations for neurosurgeons managing acute stroke patients. First, early initiation of sacubitril/valsartan for hypertension may support effective heart failure management in patients with ICH. Second, sacubitril/valsartan may be safely administered to patients with cerebral hemorrhage requiring multiple antihypertensive agents.

In the management of ICH, heart failure or reduced left ventricular function has been reported to occur in approximately 2.1% of patients within 30 days of onset [2] and represents an independent risk factor for increased mortality [8,9]. These findings highlight the importance of careful heart failure management in patients with ICH. NT-proBNP monitoring is widely recommended in domestic and international guidelines for diagnosing heart failure, assessing severity, and predicting prognosis [10]. Elevated NT-proBNP levels have also been reported in patients with ICH, with one study showing a mean admission value of approximately 400 pg/mL and a peak of 666.8 ± 355.1 pg/mL on day 4, followed by a gradual decline [11]. Several studies have demonstrated that sacubitril/valsartan therapy induces a rapid and significant decrease in NT-proBNP levels in patients with heart failure [12,13]. Although the dosing frequency of sacubitril/valsartan differs between heart failure and hypertension, once-daily dosing for hypertension has also been shown to reduce NT-proBNP levels over time in heart failure patients [14]. In our case, the NT-proBNP level was 1823 pg/mL at admission, indicating a high risk of cardiac failure during perioperative intensive care. Following the initiation of sacubitril/valsartan, a rapid decline in NT-proBNP was observed, which thereafter remained consistently low throughout hospitalization. The rapid and sustained decline in NT-proBNP levels observed in this case is unlikely to be explained solely by the natural course of cerebral hemorrhage, suggesting that early sacubitril/valsartan initiation contributed substantially to this outcome. From the perspective of heart failure management, the early initiation of sacubitril/valsartan as an antihypertensive agent in patients with ICH may help prevent complications related to cardiac dysfunction.

Strict blood pressure control is essential to prevent rebleeding in ICH, making rapid stabilization of blood pressure an important component of management [7]. Clinically, patients with ICH and refractory hypertension often present challenges when transitioning from continuous intravenous antihypertensive therapy to oral medications. Prolonged nicardipine infusion can lead to various complications [15], underscoring the need for potent oral antihypertensive agents. Although the antihypertensive effect of sacubitril/valsartan has been established, the introduction of sacubitril/valsartan in the acute phase of ICH has not been previously reported in the literature. The 2021 Japanese Stroke Guidelines limit recommended drug classes to conventional agents such as angiotensin receptor blockers and angiotensin-converting enzyme inhibitors, calcium channel blockers, and diuretics [7]. Sacubitril/valsartan is not described, and the guidelines do not address the clinical situations in which its use may be appropriate. It is generally introduced once blood pressure is stabilized and during the transition to the chronic phase. Sacubitril/valsartan has been reported to exert superior antihypertensive effects compared with olmesartan [5] and to be particularly effective in patients with severe hypertension [4]. The antihypertensive effect of sacubitril/valsartan has been reported to peak within 1 to 2 weeks after initiation, followed by a gradual decline up to week 8 [16]. On the other hand, it is also important to note that this drug carries a risk of hypotension. Excessive or abrupt reduction carries the risk of lowering cerebral perfusion pressure, potentially resulting in perihematomal ischemia and subsequent infarction [17]. Careful selection of appropriate cases may help maximize the therapeutic benefits of this medication. In our case, a combination of five oral antihypertensive agents was needed ultimately, and sacubitril/valsartan could be used without hypotension. The dose of intravenous antihypertensive agent used in this case exceeded the maximum recommended dose of a single oral antihypertensive agent [15]. The TRICE score, a tool predicting the requirement for three or more antihypertensive agents within three months after ICH (positive when ≥3 points), was six in this case, indicating a high likelihood of needing three or more agents [18]. In patients with ICH requiring multiple antihypertensive agents, sacubitril/valsartan has a low risk of hypotension and is an effective option during the transition from intravenous to oral antihypertensive therapy.

Because this is a single case report, the findings should be interpreted with caution, and the clinical course may have been influenced by factors other than sacubitril/valsartan. In addition, attention should be paid to the fact that indications for sacubitril/valsartan vary across countries and regions. Clarifying which clinical subtypes may respond favorably to sacubitril/valsartan will be an important issue for future investigation.

## Conclusions

There is currently no consensus on the optimal selection or sequencing of oral antihypertensive agents for ICH, and therapy should be individualized based on the underlying pathology. This case suggests that early initiation of sacubitril/valsartan may represent a promising therapeutic option for patients with ICH complicated by refractory hypertension and chronic heart failure, providing both effective blood pressure reduction and additional cardioprotective benefits.
